# Temperature Determination and Scene Change Artifact Mitigation When Using Fourier-Transform Spectroscopy on Targets with Time-Varying Temperature

**DOI:** 10.3390/s26082512

**Published:** 2026-04-18

**Authors:** Kody A. Wilson, Michael L. Dexter, Benjamin F. Akers, Anthony L. Franz

**Affiliations:** Center for Technical Intelligence Studies and Research, Air Force Institute of Technology, 2950 Hobson Way, Fairborn, OH 45433, USA

**Keywords:** scene-change artifact, signal-to-SCA ratio, smooth offset correction, Fourier-transform, hyperspectral imaging

## Abstract

**Highlights:**

**What are the main findings?**

- A method for temperature determination using Fourier-transform spectroscopy was developed that produces 6320 times more temperature measurements.
- A method for mitigating scene-change artifacts in Fourier-transform spectra was demonstrated for thermally variant targets.

**What are the implications of the main findings?**

- Enables retrieval of additional temperature profile information for thermally variant targets without the need for additional instrumentation.
- The accuracy of Fourier-transform spectroscopy measurements for thermally variant targets was significantly improved.

**Abstract:**

Fourier-transform spectroscopy is a widely used technique for determining the spectral and thermal properties of a target. However, target temperature variations during measurement can compromise the spectral accuracy. Temperature fluctuations induce oscillations superimposed on the target spectrum. These oscillations, referred to as scene-change artifacts, degrade the spectral accuracy. The literature is divided, with theoretical predictions suggesting negligible artifacts and growing experimental evidence reporting significant artifacts. This paper presents a theory and experimental validation of scene-change artifacts originating from target temperature variations. Traditionally, the interferogram offset is assumed to be constant, an invalid assumption for a changing scene. The error is subsequently Fourier-transformed, producing scene-change artifacts. Accurately estimating the truth spectrum is often challenging. To address this, we propose the signal-to-scene-change-artifact ratio, a metric that quantifies the impact of scene-change artifacts without knowledge of the truth spectrum. The artifacts will be eliminated by estimating the interferogram offset using smooth offset correction. Furthermore, the interferogram offset enables determination of the target’s temperature with a greater accuracy and an increased temporal resolution compared to using the spectra. These results will demonstrate that a smooth offset correction can improve the spectrum and temperature accuracy on thermally variant targets when measured with a Fourier-transform spectrometer.

## 1. Introduction

Ascertaining a target’s spectral and temperature characteristics is a prevalent challenge in scientific analyses [1]. Spectral measurements are used to characterize the target, quantify chemical emissions, classify materials, and identify detonation sources [2,3,4]. Temperature measurements provide insight into combustion diagnostics and energy balancing [5,6].

Dispersive spectroscopy determines the spectrum by separating light into spectral bands using prisms or diffraction gratings. The spectral bands are measured by a detector array or a scanning detector [7]. Dispersing the light can limit the dispersive system’s spectral resolution, signal strength and measurement rate. Fourier-transform spectroscopy (FTS), or Fourier-transform infrared spectroscopy (FTIR) in the infrared (IR), provides advantages in these areas [8]. Owing to these advantages, FTS has become widely adopted [9]. FTS interferometers produce an interferogram from the incoming light. The interferometer splits incoming light into two beams that follow different paths. The two beams are recombined and undergo constructive or destructive interference depending on the optical path difference (OPD) [10]. The OPD is controlled by an adjustable mirror placed on one of the paths. The measured interferogram is the signal recorded as the mirror scans through OPDs. The scan of the Telops Hyper-Cam used in this study ranges from seconds to minutes [11]. Although suitable for stable targets, temperature variations during this period can affect the spectral accuracy [12]. These scene changes introduce artifacts known as scene-change artifacts (SCAs) across the spectrum [13].

SCAs often appear as multi-frequency oscillations in the spectrum and are commonly considered noise [14]. SCAs are superimposed on the measured spectrum. Kick theoretically showed that the measured spectrum corresponds to the spectrum observed at the zero-path difference (ZPD) [15]. Kick addressed the measured spectrum when the field of view (FOV) was changed, but similar results were seen when simulating targets with variable temperatures [16]. SCAs can be grouped into four types: oscillatory, stochastic, FOV and temperature changes. Oscillatory scene changes occur when the target is periodically obscured. Examples of oscillatory scene changes include viewing the target through clouds or a chopper wheel [17,18]. The measured interferogram exhibits clear oscillations due to the alternation between the target and the obscuration [19]. In the spectrum, oscillatory SCAs are characterized by a central large peak with smaller harmonics at wavenumbers associated with the chopper wheel frequency [20]. Stochastic SCAs result from random target variations, such as engine exhaust. Random variations dominate the measured interferogram, with the normal interferogram pattern recognizable only near the ZPD. In the spectrum, stochastic SCAs manifest as random fluctuations, resembling a low signal and high noise [21]. FOV scene changes occur due to targets entering or exiting the FOV. The measured interferogram varies linearly with the brightness of targets filling the FOV. In the spectrum, FOV SCAs appear as oscillations around the ZPD spectrum [22]. Temperature scene changes occur when the target temperature varies, such as during combustion. This paper investigates SCAs caused by temperature variability. The measured interferogram exhibits a non-linear dependence on the target’s temperature. Variable temperature SCAs appear as oscillations around the ZPD spectra, similar to those observed from FOV SCAs.

Variable temperature SCAs will be shown to originate from incorrect interferogram offset estimation. It is typically assumed that the interferogram offset is constant, an assumption that is valid for a stable scene [23]. This assumption is invalid when the scene changes. The difference between the assumed constant and true interferogram offset is Fourier-transformed, resulting in SCAs in the spectrum. Previous authors have focused on the interferogram without offset, overlooking the errors in interferogram offset estimation, leading them to underestimate the magnitude of SCAs [24]. These predictions were used to justify disregarding SCAs in the measured spectra [25].

Oscillatory SCAs are mitigated with the recombobulation correction method (RCM), which reconstructs the target interferogram [20]. Stochastic SCAs are mitigated using statistical methods [26,27]. Unfortunately, both approaches require the acquisition of multiple interferograms to be effective. FOV SCAs are mitigated with smooth offset correction (SOC), which estimates the variable interferogram offset [22]. SOC estimates the interferogram offset by smoothing the measured interferogram with locally weighted scatter plot smoothing (LOWESS) [28]. A single interferogram is sufficient for SOC to be effective. This paper extends this work by investigating the SCAs caused by temperature variations occurring over the interferogram scan time. These variations cause nonlinear interferogram offset changes as compared to the linear offset changes caused by FOV variations. The previously developed SOC method is applied to the nonlinearly varying interferogram offsets to determine its effectiveness. If effective, SOC would mitigate SCAs from multiple scene-change scenarios without the need to distinguish them first.

In addition to correcting SCAs, the interferogram offset estimate can be used to determine the target’s temperature. Under standard FTS procedures, the target temperature is determined by analyzing the resulting spectrum. This technique involves integrating over a spectral band to determine the target’s brightness temperature. Kick’s results indicate that this approach yields only the target’s temperature at the ZPD. SOC estimates the interferogram offset at every OPD, which can then be used to determine the target’s temperature at every OPD. A single interferogram can contain thousands of measurements. This produces a temperature profile across the interferogram, rather than a single temperature value.

In this paper, the relationship between the target temperature and the interferogram offset is examined first. Next, the procedure for simulating interferograms for a target spectrum is outlined. Interferograms for several blackbody temperatures are simulated. The calibration procedure for determining the temperature is demonstrated. SOC is applied to the interferograms to estimate the interferogram offset. The interferogram offset is then used to estimate the blackbody temperatures. Modifications to the calibration procedures associated with the detector bandwidth are demonstrated.

The standard FTS processing methodology is reviewed. During this review, a theoretical basis for SCAs in the calibrated spectrum is developed. Then, the steps for simulating an interferogram with a variable temperature target are examined. Temperature SCAs under a variety of conditions are presented. SOC is demonstrated, removing nearly all the temperature SCAs. Two metrics for estimating SCA significance are presented. The normalized root mean square error (NRMSE) compares the calibrated spectrum to the truth spectrum [29,30]. We propose a new metric, the signal-to-SCA ratio (SSR), to quantify the relative magnitude of the signal and the SCAs. The SCA magnitude scales with the temperature change, detector bandwidth, and maximum optical path difference (MOPD). SOC is shown to improve the NRMSE and SSR for a variety of SCA conditions.

The experiment began by measuring a blackbody using a Telops Hyper-Cam LW, an FTS hyperspectral imager. Calibration was performed using measurements of blackbodies at various temperatures. The accuracy of temperature determination using the SOC interferogram offset estimate was evaluated with a constant temperature blackbody. Then, a steady-state and variable temperature glow bar was measured. Having validated the accuracy of temperature determination, the same method was applied to the glow bar as it heats. The measured interferograms from the heating glow bar were processed traditionally and with SOC for comparison. The improvement was quantified using the NRMSE and SSR for the calibrated and raw spectra. SCAs were the largest, and SOC provided the most improvement, when there was a large temperature difference between the start and end of the interferogram.

## 2. Theory and Simulation

### 2.1. FTS Temperature Estimation Theory

Consider how the measured interferogram can be used to determine the target’s temperature. Assume an incoming blackbody spectrum given by:

(1) $$L_{BB}\left(\nu ,T\right)=2{hc}^{2}\nu^{3}\frac{1}{e^{\frac{hc\nu }{kT}}-1},$$

where h is Planck’s constant, c is the speed of light, ν is the wavenumber, k is Boltzmann’s constant, and T is the temperature [14,31]. When this spectrum is incident on a detector, it is converted into an electrical signal that is subsequently recorded by the electronics. The uncalibrated spectrum is given by:

(2) $$L_{\mathrm{uncal}}\left(\nu \right)\;=\;G\left(\nu \right)\left[L(\nu )\;+\;O\left(\nu \right)\right]$$

where G is the calibration gain, O is the calibration offset, and L is the incoming spectrum [32].

Prior to reaching the detector, the incoming spectrum passes through an interferometer, where the beam is split into two paths of different lengths before being recombined [33]. A movable mirror changes the distance one beam travels, creating different optical path differences (OPDs). The OPD represents the difference in the optical paths traveled by the two beams in the interferometer. The measured interferogram is the signal recorded at the detector as a function of the OPD. The measured interferogram includes two components and is given by:

(3) $$I_{m}{\left(x\right)\;=\;I(x)\;+\;I}_{o},\;$$

where I(x) is the interferogram without offset, $I_{0}$ is the interferogram offset and x is the optical path difference (OPD) [34]. For a constant input spectrum, the interferogram offset is constant.

An early step in FTS processing is the removal of the interferogram offset [35]. However, the interferogram offset contains valuable information related to the target’s temperature. The interferogram offset is equal to half the measured interferogram at the ZPD [36].

(4) $$I_{o}=\frac{1}{2}\int_{0}^{\infty }G\left(\nu \right)\left[2{hc}^{2}\nu^{3}\frac{1}{e^{\frac{hc\nu }{kT}}-1}+O\left(\nu \right)\right]d\nu .$$

For an instrument with a constant calibration gain and zero calibration offset, the integral of the spectral radiance becomes the total radiance.

(5) $$I_{o}=\frac{1}{2}{GL}_{total},$$

where G is a constant calibration gain and $L_{total}$ is the total radiance over all wavenumbers. Assuming a Lambertian surface, the total radiance can be related to the total exitance by:

(6) $$L_{total}=\frac{M_{total}}{\pi }$$

where $M_{total}$ is the total exitance [37]. The total exitance of a blackbody is given by the Stefan–Boltzmann law:

(7) $$M_{total}=\sigma T^{4},$$

where σ is the Stefan–Botlzmann constant and T is the temperature [38]. This equation assumes integrations over all wavenumbers; the finite bandwidth of the FTS detector will be considered below. Combining Equations (4)–(7) yields a relationship between the interferogram offset and temperature, given by:

(8) $$I_{o}=\frac{G}{2}\frac{\sigma }{\pi }T^{4}.$$

Solving for temperature yields:

(9) $$T=\sqrt[{4}]{\frac{2}{G}\frac{\pi }{\sigma }I_{o}}$$

This result suggests that the target temperature can be determined using the interferogram offset.

Under stable conditions, the interferogram without offset contains the same temperature information. However, if the target’s temperature changes, during the interferometer mirror scan, the interferogram offset varies accordingly. This variation generates SCAs in the measured spectra, which degrade the temperature determination. No temperature accuracy is lost when using the interferogram offset to determine the temperature in the presence of temperature fluctuations. By combining all constants from Equation (9), a simple relationship can be derived between the target temperature and the interferogram offset.

(10) $$T\left(x\right)=C\;I_{o}^{\frac{1}{4}}\left(x\right)$$

where C is the temperature calibration constant that incorporates the terms from Equation (9) and is determined empirically by measuring a small set of targets with known temperatures. This approach is similar to the procedure used to determine the calibration gain in Equation (2) discussed in Section 2.3. As such, this methodology does not require significant additional effort from the experimenter. A single measured interferogram can contain thousands of data points. Estimating the temperature at each OPD provides the temporal temperature profile of the target rather than the single temperature estimate given by the spectrum.

### 2.2. FTS Temperature Estimation Simulation

To model the effectiveness of estimating the temperature at every OPD, several measured interferograms were simulated. Eight interferograms without offset were simulated for blackbody temperatures ranging from 323 K to 651 K (50 °C to 378 °C) at 50 K increments, with a smaller final increment. These temperatures mirror those used in the experiments conducted later to validate these simulations. Blackbody spectra were simulated using Equation (1) at a 1 cm^−1^ spectral resolution. Then, the blackbody spectra were substituted into Equation (2). Initially, a uniform calibration gain and offset were applied for all wavenumbers. Subsequently, a calibration gain that is flat within the LWIR (861 cm^−1^ to 1306 cm^−1^) and zero outside the LWIR was used in Equation (2) to demonstrate the adjustments necessary for a non-ideal instrument. This spectral range was selected to match the performance of the instrument used during the experiment. The raw spectra were substituted into Equation (11) to retrieve the interferograms without offset.

(11) $$I\left(x\right)=\frac{1}{2}I^{-1}\left\{L_{uncal}\left[\nu ,T\right]+L_{uncal}\left[-\nu ,T\right]\right\},$$

where $I^{-1}$ denotes the inverse Fourier-transform [39]. The inverse and forward Fourier-transforms were implemented using zero-filled inverse fast Fourier-transforms (IFFT) and fast Fourier-transforms (FFT), respectively [40]. Based on the 1 cm^−1^ resolution of the original spectrum, the interferogram had an MOPD of 0.5 cm and was composed of 20,000 measurements. The interferogram offset is equal to the maximum of the interferogram without offset, which occurs at the ZPD and is given by:

(12) $$I_{o}=I\left(0\right).$$

**Figure 1:**
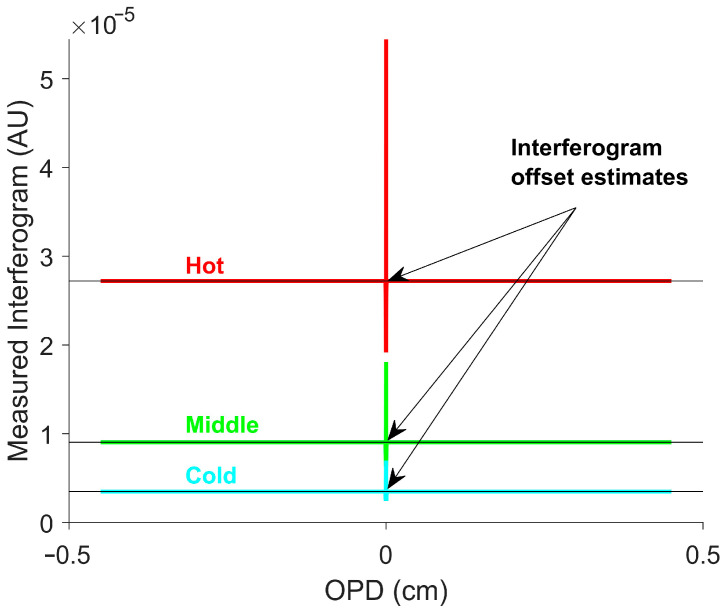
Simulated measured interferograms for three blackbody temperatures. The red, green, and blue lines represent the hot, middle, and cold temperatures of 623 K, 473 K, and 373 K (350 °C, 200 °C, 100 °C), respectively. The black lines indicate the interferogram offset estimates for each simulated measured interferogram. The interferogram offset estimate is flat, including near the ZPD.

Equations (11) and (12) were then substituted into Equation (3) to generate the eight simulated measured interferograms for the blackbody targets. Three simulated measured interferogram examples are shown in Figure 1 [41].

**Figure 2:**
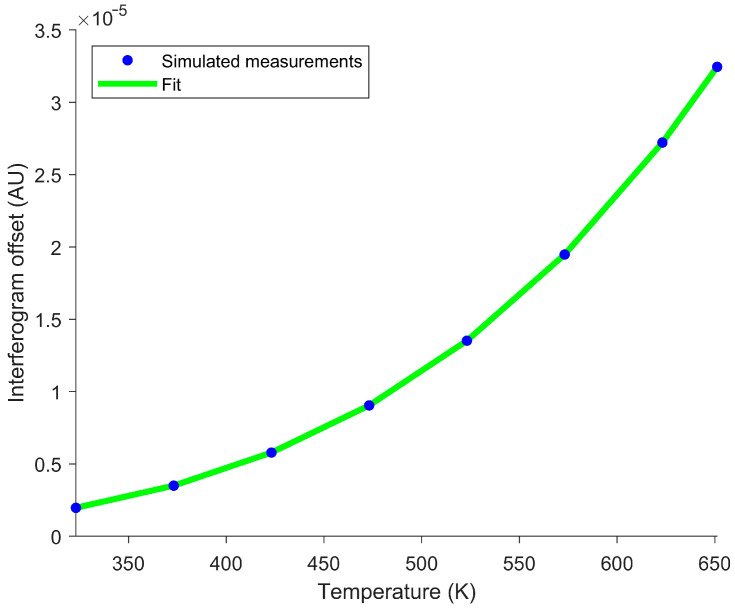
Simulated interferogram offset averages for the eight blackbody temperatures. Blue circles represent the calculated interferogram offset, while the green curve shows the associated $T^{4}$ curve fit predicted by Equation (9). The good agreement of the curve fit suggests that, when an interferogram offset is determined, it can be used to estimate the target’s temperature.

The interferogram offset was determined using SOC. SOC was previously shown to be effective in estimating the interferogram offset [22]. SOC does a moving average over the window, but weighs the center of the window more than points near the edge of the window [28]. This is well suited for smoothing around the ZPD; by the time a peak has a large weight, it will be moderated by valleys preventing the large variations near the ZPD seen with other smoothing techniques. This technique is particularly easy to implement, since LOWESS is a built-in option in MATLAB R2024B’s “smoothdata” function with the “lowess” option [42]. Large windows increase the accuracy near the ZPD, while small windows increase the accuracy for fast temperature changes. The window size used was 400. The average interferogram offset across all OPDs for each simulated blackbody temperature is shown in Figure 2.

**Figure 3:**
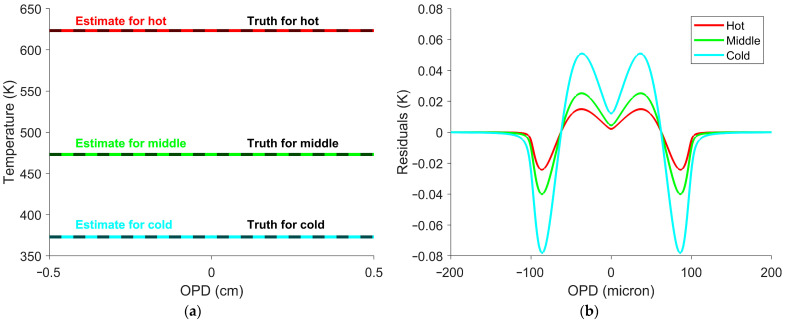
Temperature estimates and residuals from simulated measured interferograms. (**a**) The red, green, and blue lines represent the hot, middle, and cold temperatures of 623 K, 473 K, and 373 K (350 °C, 200 °C, and 100 °C), respectively. Each estimate is compared with the truth temperature shown in black. (**b**) Residuals between the estimated and truth temperatures, plotted only near the ZPD. The OPDs shown represent a small portion (0.4%) of the full OPD range. The temperature estimates are accurate across the interferogram, with the largest deviations of less than 0.1 K occurring only near the ZPD.

This simulated dataset was used to determine the temperature calibration constant in Equation (10) in a manner analogous to how it would be determined experimentally using multiple blackbody measurements. Once the temperature calibration constant was obtained, Equation (10) was used to estimate temperature at every OPD position shown in Figure 1. Figure 3 shows the resulting temperature profiles and associated errors.

The largest inaccuracies were observed only near the ZPD, with Figure 3b representing just 0.4% of all the OPDs. Away from the ZPD, the estimated temperature deviated by no more than $3\times 10^{-5}$ K from the true value. Increasing the SOC window size above 400 points, equivalent to a 0.02 cm OPD, further smooths the variation at the ZPD. Smaller window sizes better estimate interferogram offsets from faster variations, but at the cost of accuracy near the ZPD. The window size can be decreased to 100 points, equivalent to a 0.005 cm OPD, before the temperature error at the ZPD reaches 1 K. This indicates that an FTS can be used to determine the target’s temperature at every OPD, but will smooth fast temperature variations.

**Figure 4:**
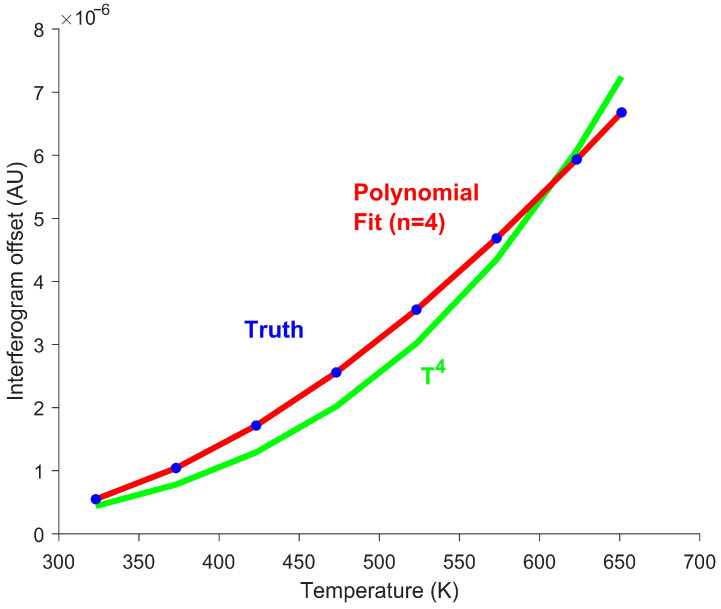
Simulated interferogram offsets for blackbody temperatures observed with a limited bandwidth. Blue circles represent the simulated interferogram offset values. The green curve shows the associated $T^{4}$ fit, which deviates from the truth values due to the limited spectral range. The red curve shows a fourth-degree polynomial fit, which closely matches the simulated data. The polynomial fit better matches the measurements from the non-ideal detector. The polynomial fit also requires more calibration measurements to generate the fit than the power fit used for the ideal detector.

Although assuming infinite limits for the observed wavenumbers is analytically convenient, this assumption is unrealistic. In practice, a spectrometer cannot measure the full spectrum because of its finite spectral bandwidth. Consequently, the Stefan–Boltzmann law, which requires integration over the full blackbody spectrum, is no longer applicable. To account for this limitation, assume the gain function in Equation (4) is flat, but limited to a finite spectral range. Under this assumption, Equation (4) can be rewritten as:

(13) $$I_{o}(T)={hc}^{2}G\int_{\nu_{1}}^{\nu_{2}}\nu^{3}\frac{1}{e^{\frac{hc\nu }{kT}}-1}d\nu$$

where $\nu_{1}$ and $\nu_{2}$ are the lower and upper wavenumber limits, respectively. Notably, this integral has no known analytical solution [43]. For blackbody temperatures of 623 K, 473 K, and 373 K (350 °C, 200 °C, and 100 °C), less than a third of the radiant energy lies within the longwave infrared (LWIR) between 861 cm^−1^ and 1306 cm^−1^ [44]. Importantly, the fraction of the observed energy varies with the temperature. This variability violates the $T^{4}$ proportionality inherent in the Stefan–Boltzmann law, causing inaccuracies in temperature estimation when the full spectrum is not observed, as shown in Figure 4.

**Figure 5:**
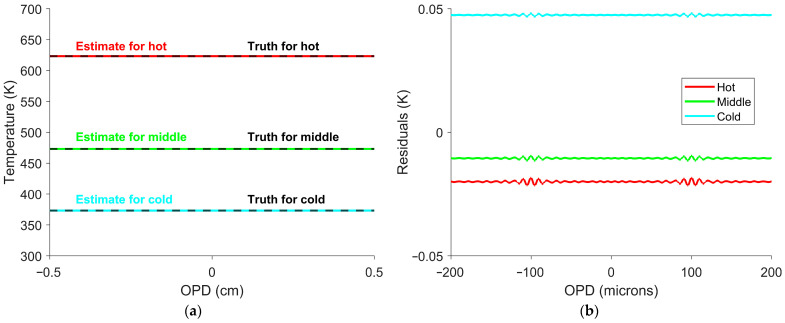
Temperature estimates at each OPD for a simulated blackbody measured interferogram with a limited bandwidth. (**a**) Red, green, and blue lines represent temperature estimates at each OPD for blackbody temperatures of 623 K, 473 K, and 373 K (350 °C, 200 °C, and 100 °C), respectively. The black dotted lines indicate the true blackbody temperatures. (**b**) Residuals between the true temperature and the estimated temperatures. Estimating the temperature with the SOC interferogram offset is accurate for non-ideal detectors with minor alterations to the methodology.

To account for the limited spectral bandwidth, a fourth-degree polynomial fit was used to fit the interferogram offset vs. temperature curve. Figure 4 demonstrates that this polynomial more accurately represents the interferogram offset than the $T^{4}$ fit. In order to determine the polynomial fit, additional calibration measurements of a known temperature target are required. Additionally, solving for temperature using the polynomial fit produces four potential solutions. Only one solution is physically meaningful. The other three are discarded as non-physical. The remaining valid solution is the temperature estimate. This approach provides highly accurate temperature estimates, as demonstrated in Figure 5.

Despite the limited spectral range, the error remains low within 0.05 K across the entire interferogram. This simulation suggests that the temperature can be accurately estimated at every OPD position for non-ideal detectors.

### 2.3. SCA Temperature Transition Theory

The interferogram offset in Equation (3) provides valuable temperature information. Next, we analyzed the interferogram without offset. The interferogram without offset was isolated from the interferogram offset. The interferogram offset is traditionally estimated by computing the average of the measured interferogram. This estimate is subtracted from the measured interferogram to obtain the interferogram without offset.

If the target’s temperature is constant, the traditional method accurately removes the interferogram offset. However, if the target’s temperature varies, the interferogram offset varies accordingly. In these cases, a constant interferogram offset is an invalid assumption. Traditional processing produces an inaccurate estimate of the interferogram without offset, given by:

(14) $$I_{est}\left(x\right)=a\left(x\right)\left\{I\left(x\right)+[I_{o}(x)-I_{o,est}]\right\},$$

where a(x) is the apodization or truncation function applied to the interferogram and $I_{o,est}$ is the estimated interferogram offset. The estimated interferogram without offset is then Fourier-transformed. The resulting spectrum is called the raw spectrum [45]. The raw spectrum can be expressed as:

(15) $$L_{raw}\left(\nu \right)=I[a\left(x\right)I(x)]+I\left\{a\left(x\right)\left[I_{o}(x)-I_{o,est}\right]\right\}.$$

Even under ideal conditions, truncation of the interferogram limits the spectral resolution, introduces oscillations near sharp features, and leads to discrepancies between the raw and uncalibrated spectra [46].

When a temperature change is present in the scene, the first term in the raw spectrum, in Equation (15), corresponds to the spectrum observed at the ZPD. SCAs appear due to the second term, the Fourier-transform of the interferogram offset error.

(16) $$L_{raw}\left(\nu \right)=L_{raw,ZPD}\left(\nu \right)+L_{raw,SCA}\left(\nu \right)$$

Subsequently, the raw spectrum is substituted into Equation (2) in place of the uncalibrated spectrum, and the expression is solved for the target spectrum. This result is referred to as the calibrated spectrum, given by:

(17) $$L_{cal}\left(\nu \right)=\frac{L_{raw,ZPD}\left(\nu \right)}{G_{est}\left(\nu \right)}-O_{est}\left(\nu \right)+\frac{L_{raw,SCA}\left(\nu \right)}{G_{est}\left(\nu \right)}$$

where $G_{est}$ and $O_{est}$ are the estimated calibration gain and offset, respectively, given by:

(18) $$G_{est}\left(\nu \right)=\frac{L_{hot,raw}\left(\nu \right)-L_{cold,raw}\left(\nu \right)}{L_{hot}\left(\nu \right)-L_{cold}\left(\nu \right)}$$

and:

(19) $$O_{est}\left(\nu \right)=\frac{L_{cold,raw}\left(\nu \right)L_{hot}\left(\nu \right)-L_{hot,raw}\left(\nu \right)L_{cold}\left(\nu \right)}{L_{hot,raw}\left(\nu \right)-L_{cold,raw}\left(\nu \right)},$$

where $L_{hot,raw}(\nu )$ and $L_{cold,raw}(\nu )$ are the raw spectra of the hot and cold reference blackbodies, respectively, while $L_{hot}\left(\nu \right)$ and $L_{cold}\left(\nu \right)$ are the corresponding known spectra [47,48]. Complex values in the calibrated spectrum, gain, and offset arise from asymmetries in the measured interferograms. Traditionally, these asymmetries result from phase shifts and random noise within the interferometer and detector. In this analysis, only the real component of the calibrated spectrum was considered, while the imaginary component was discarded. Kick predicted significant SCAs in the imaginary component and negligible SCAs in the real component [15]. Based on Kick’s prediction and discarding the imaginary component, previous authors justified ignoring SCAs in the calibrated spectra [49]. This work demonstrates that SCAs are generated from a target with a changing temperature.

Improving the estimation of the interferogram offset can eliminate SCAs in the calibrated spectrum. To accomplish this, the interferogram offset was determined by applying smooth offset correction (SOC) to the measured interferogram. As a result, the calibrated spectrum accurately represents the ZPD spectrum.

### 2.4. SCA Temperature Transition Simulation

To illustrate the impact of SCAs, a target with a time-varying temperature profile was simulated. Assume that a target’s temperature changes linearly with time, defined by:

(20) $$T\left(t\right)=C_{t}t+T_{0},$$

where $C_{t}$ is the temperature change rate, $T_{0}$ is the initial temperature, and t denotes time. The OPD depends on time and the mirror velocity, given by:

(21) $$x(t)=2vt-x_{MOPD},$$

where v is the mirror velocity and $x_{MOPD}$ is the maximum optical path difference (MOPD) [50]. At t=0 the target will be at $T_{0}$ and the interferometer’s OPD will be $-x_{MOPD}$. Combining Equations (20) and (21) gives the temperature as a function of the OPD:

(22) $$T\left(x\right)=\frac{C_{t}}{2v\left(x+x_{MOPD}\right)}+T_{0}.$$

A double-sided interferogram with an MOPD of 0.777 cm and 8192 OPD positions was used for the simulations. The target’s temperature was computed from Equation (22) for each OPD. The measured interferogram for a blackbody at that temperature was simulated using Equations (11) and (12). From the simulated measured interferogram, a single data point was extracted to represent the expected measurement at the specified temperature and OPD. Repeating this procedure for all OPDs produces the measured interferogram for a target undergoing a linear temperature transition. This methodology is easily adapted to more challenging thermal behaviors, such as an exponentially varying temperature. Minor modifications to the derivation of Equation (22) are sufficient to incorporate the new temperature transition.

**Figure 6:**
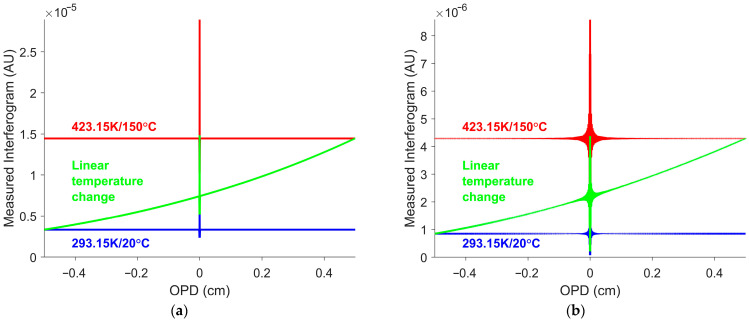
Simulated measured interferograms for a linear temperature transition from 293 K to 423 K (20 °C to 150 °C). (**a**) Uniform gain model. (**b**) LWIR gain model. The green curves represent the measured interferograms corresponding to the linear temperature transition. The red and blue curves correspond to the measured interferogram of blackbodies with temperatures of 423 K and 293 K (150 °C and 20 °C), respectively. The interferogram offset can no longer be represented by a constant. Note that, despite the linear temperature change, the interferogram offset is non-linear.

The simulations were performed with the target temperature varying linearly from 293 K to 423 K (20 °C to 150 °C). Two sets of calibration gains and offsets were applied. The first model applied a uniform calibration gain across all wavenumbers, representing an ideal instrument capable of measuring the full spectrum. The second model applied a calibration gain of one in the LWIR region (861 to 1306 ${cm}^{-1}$), and zero outside this region, simulating a detector with a narrow spectral response. For both models, the calibration offset was assumed to be zero for simplicity. The measured interferograms for the two simulations are shown in Figure 6.

The measured interferograms exhibited non-linear behavior, despite the linear temperature transition. This occurred because the interferogram offset does not vary linearly with temperature. Comparison of the y-axis scales indicated that the measured interferogram in Figure 6a (uniform gain) had a larger magnitude than that in Figure 6b (LWIR gain). Because the LWIR detector measures only LWIR signals, its interferogram offset was smaller.

**Figure 7:**
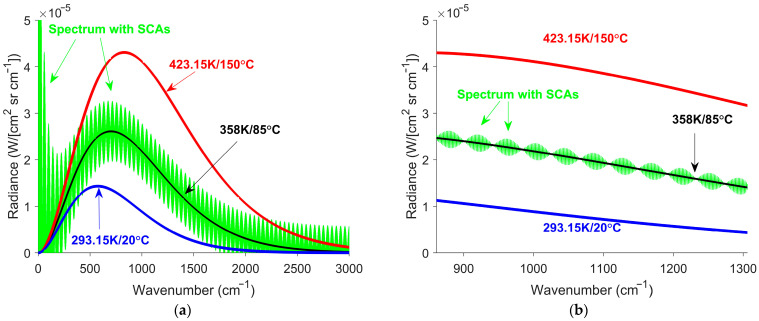
Simulated calibrated spectra with SCAs for linear temperature transitions. (**a**) Uniform gain model. (**b**) LWIR gain model. The green curves represent calibrated spectra obtained via traditional processing. The black curves indicate the ZPD spectra. The red and blue curves correspond to the calibrated spectra of blackbodies with temperatures of 423 K and 293 K (150 °C and 20 °C), respectively. Each case has SCAs superimposed on the ZPD spectra, but the LWIR SCAs are smaller.

A hot blackbody, 473 K (200 °C), and a cold blackbody, 323 K (50 °C), were simulated for calibration. Although such blackbodies typically span a broader temperature range than the expected targets, the selected values were constrained by the experimental setup discussed in Section 3. The calibration gain and offset were determined using the simulated interferograms of the hot and cold blackbodies. The measured interferograms in Figure 6 were processed using the traditional approach. The interferogram offset estimate was obtained by averaging the measured interferogram. Subtracting the estimate from the measured interferogram yielded the interferogram without offset. The interferogram without offset was then Fourier-transformed to obtain the raw spectrum. The raw spectrum was calibrated to produce the calibrated spectra shown in Figure 7.

Significant SCAs were evident in the calibrated spectra shown in green in Figure 7. The SCAs were noticeably smaller in Figure 7b than in Figure 7a. The decrease is attributed to the smaller interferogram offset in Figure 6b compared with Figure 6a.

Smaller interferogram offsets produce smaller estimation errors and, consequently, smaller SCAs. A similar effect is expected for smaller temperature transitions. Smaller transitions reduce interferogram offset estimation errors, resulting in smaller SCAs. When the temperature difference approaches zero, SCAs vanish, as expected for FTS observing steady-state conditions. The simulation used an idealized sharp cutoff for the calibration gain. For a real detector, the calibration cutoff is more gradual. Consequently, a signal outside the detector’s specified response region still contributes to the interferogram offset and SCAs.

**Figure 8:**
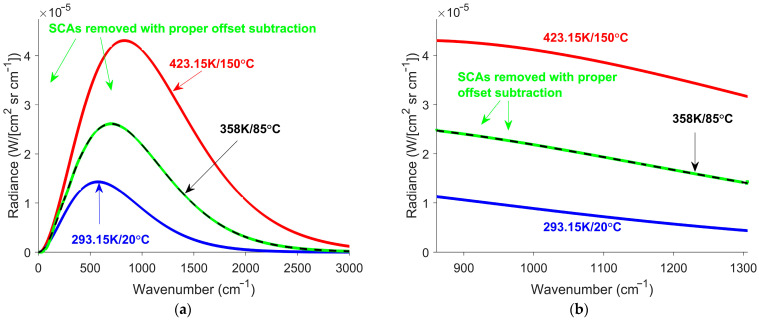
Simulated corrected spectra for a linear temperature transition for the (**a**) uniform gain model and (**b**) LWIR gain model. The green curves represent the calibrated spectra after SOC correction. The black curves indicate the ZPD spectra. The red and blue curves correspond to the calibrated spectra of blackbodies with temperatures of 423 K and 293 K (150 °C and 20 °C), respectively. SOC was effective at mitigating SCAs for both cases.

To mitigate the SCAs in Figure 7, the interferogram offset was estimated using smooth offset correction (SOC) [20]. Using the improved interferogram offset estimate removes the primary source of SCAs identified in Equation (15). Figure 8 shows the calibrated spectra after correction.

The corrected spectra resemble the ZPD spectra. The improvement was quantified by comparing the calibrated spectra before and after correction to the ZPD spectra. The normalized root mean square error (NRMSE) was calculated for each spectrum using:

(23) $$\mathrm{NRMSE}=\frac{1}{n^{2}\{\max\left[L_{\mathrm{ZPD}}(\nu )\right]-\min\left[L_{\mathrm{ZPD}}(\nu )\right]\}}\sqrt{\sum_{i=1}^{n}{\left\{Re[L_{cal}(\nu_{i})]-L_{\mathrm{ZPD}}\left(\nu_{i}\right)\right\}}^{2}},$$

where $max[L_{ZPD}(\nu )]-min\left[L_{ZPD}\left(\nu \right)\right]$ denotes the range of the ZPD spectrum [29,30]. L($\nu_{i}$) and $L_{ZPD}(\nu_{i})$ are the measured and ZPD spectra at wavenumber $\nu_{i}$, and n is the total number of spectral points. In the LWIR case shown in Figure 8b, the NRMSE was computed using only the wavenumber within the response region. The same analysis was applied to exponential temperature transitions, and a static case. A summary of the results is provided in Table 1.

The NRMSE improved in all transition scenarios. The NRMSE remained low when the correction was applied to the static scenario. The uniform model exhibited larger SCAs than the LWIR model. All transition scenarios exhibited an improvement after correction. This indicates that SOC is effective at mitigating temperature transition SCAs. SOC has been shown to effectively mitigate FOV transition SCAs in prior studies [22]. Thus, SOC provides a broadly applicable approach for mitigating SCAs.

For the no-transition case, a slight increase in error was seen. For this case, the interferogram offset was constant and the constant estimate was more accurate than the SOC estimate. If the target is unchanging and a constant interferogram offset can be assumed, then the traditional process has a slight edge in performance. But if the target changes, the SOC correction performs significantly better than the traditional method.

The NRMSE effectively quantifies SCAs only when the truth spectrum is known. The signal-to-SCA ratio (SSR) quantifies SCAs when the truth spectrum is unknown. The raw spectrum is expected to vanish outside the detector response region. SCAs inject a signal outside the spectral band that the detector can physically collect. A signal outside this region is attributed to SCAs. The SSR is defined as the ratio of the in-band to out-of-band signals, given by:

(24) $$SSR=\frac{\sum_{In}\frac{1}{N}\left|Re\left[L_{raw}\left(v_{i}\right)\right]\right|}{\sum_{Out}\frac{1}{M}\left|Re\left[L_{raw}\left(v_{k}\right)\right]\right|}$$

where N and M are the numbers of in-band and out-of-band measurements. $L_{raw}\left(\nu_{i}\right)$ is the raw spectrum at wavenumber $\nu_{i}$. SSR uses the raw spectrum because the gain estimate in Equation (18) tends toward zero outside the detector response region, causing large variability in the calibrated spectrum. The raw spectrum offers a more reliable measure of SCAs than the calibrated spectrum.

**Figure 9:**
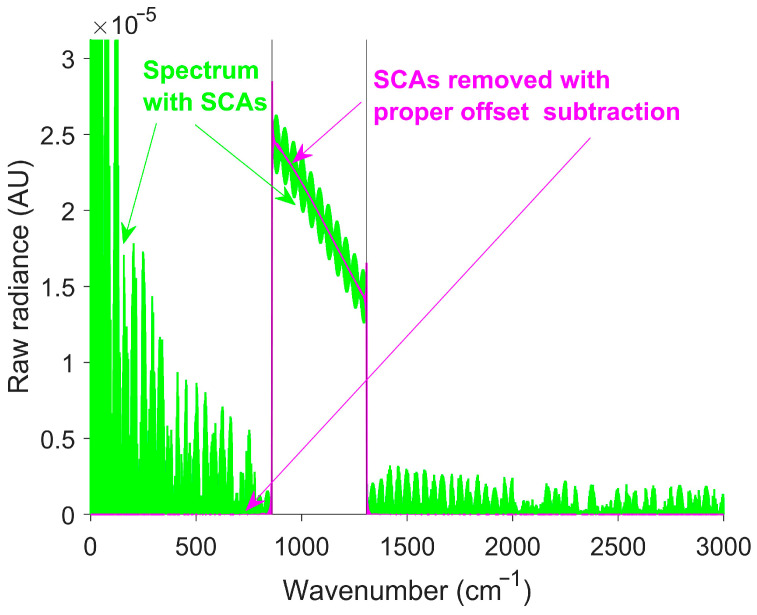
Simulated raw spectra before and after SCA correction for a linear temperature transition. The green curve represents the raw spectra before SCA correction. The magenta curve represents the raw spectra after SCA correction. The black vertical lines at 861 cm^−1^ and 1306 cm^−1^ indicate the boundaries of the response region. SOC is effective at removing SCAs from the raw spectrum, shown by the magenta line returning to zero outside the detector response region.

The raw spectrum, shown in green in Figure 9, contained a significant signal outside the calibration gain region, resulting in a low SSR. Most SCAs were removed by correction, increasing the SSR. A residual out-of-band signal occurred mainly at the edges of the response region, due to Gibbs phenomena [51]. Table 2 presents a comparison of the SSR before and after SCA correction for both the simulated linear and exponential temperature transitions.

The SSR increased markedly for both linear and exponential transitions after the correction, in agreement with the Table 1 results. In the absence of a temperature transition, the correction did not decrease the SSR. Oscillations near the gain region boundaries remained even after the SCAs were removed, due to interferogram truncation. The results show that the correction is effective in dynamic scenes and preserves the accuracy under steady-state conditions.

The simulations show that temperature transitions introduce significant SCAs in the calibrated spectrum. These SCAs primarily originate from inaccurate interferogram offset estimation. Correcting the interferogram offset effectively removes the SCAs. The findings will next be validated experimentally.

## 3. Materials and Methods

**Figure 10:**
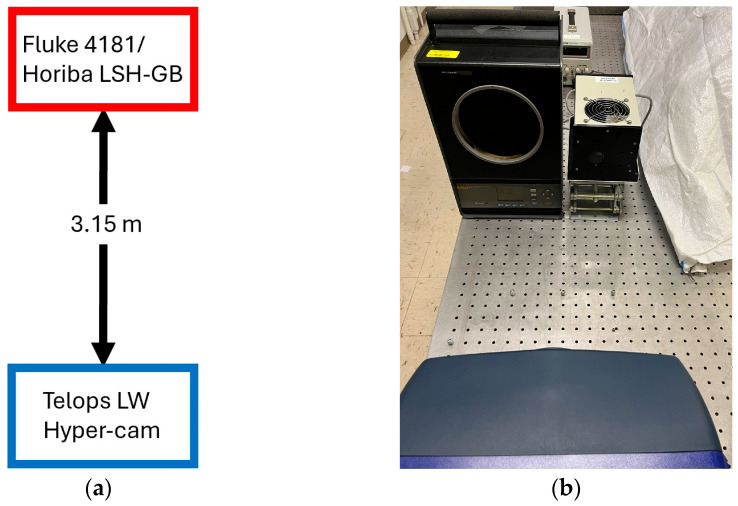
(**a**) Diagram detailing the location of equipment during the experiment. The Fluke 4181 and the Horriba LSH-GB were placed 3.15 m from the Telops LW Hyper-Cam. (**b**) Photograph of the experimental setup. The Fluke 4181 and Horriba LSH-GB were placed side by side and moved closer for the photograph. For the experiment, only one was present for each measurement. The top of the Telops LW Hyper-Cam is seen at the bottom of the picture.

Double-sided interferograms were collected using a Telops LW Hyper-Cam. The Telops Hyper-Cam LW (Telops, Quebec City, QC, Canada) operates in the LWIR between 861 cm^−1^ and 1306 cm^−1^ [52]. The interferograms were acquired with a spectral resolution of 1 cm^−1^, a spatial resolution of 128 × 256 and a 50 μs integration time. The acquisition time ranged from sub-second to greater than one minute, depending on the spectral and spatial resolutions. For these settings, the acquisition time for each interferogram was 12.4 s [53]. A Fluke 4181 precision IR calibrator (Fluke, Everett, WA, USA) was positioned 3.15 m away from the Hyper-Cam as shown in Figure 10. The Fluke 4181 was used as the reference blackbody for calibration. Eight Fluke temperature setpoints were measured from 323 K to 651 K (50 °C to 378 °C) in nominal 50 K increments, with a smaller final step [54]. The Fluke 4181 measurements were used to determine the calibration gain, offset, and temperature calibration constant from Equation (10). On 10 September 2025, the experiment was conducted in an indoor laboratory environment and lasted approximately four hours. Assuming constant atmospheric transmission and path radiance, these factors were incorporated into the calibration gain, offset and temperature calibration constant [55].

The Fluke 4181 was then replaced with a Horiba LSH-GB illuminator (Horiba Instruments, Irvine, CA, USA). The device heats a glow bar with electrical current [56]. The illuminator voltage varied from 1 V to 8 V in 1 V increments. At each voltage, ten FTS hypercubes were acquired after the glow bar reached a steady-state temperature. Next, the glow bar cooled before applying 10 V. FTS hypercubes of the glow bar were acquired while it heated. The procedure was then repeated for applied voltages of 1 V to 10 V at 1 V increments.

## 4. Results

### 4.1. FTS Temperature Estimation Experimental Results

**Figure 11:**
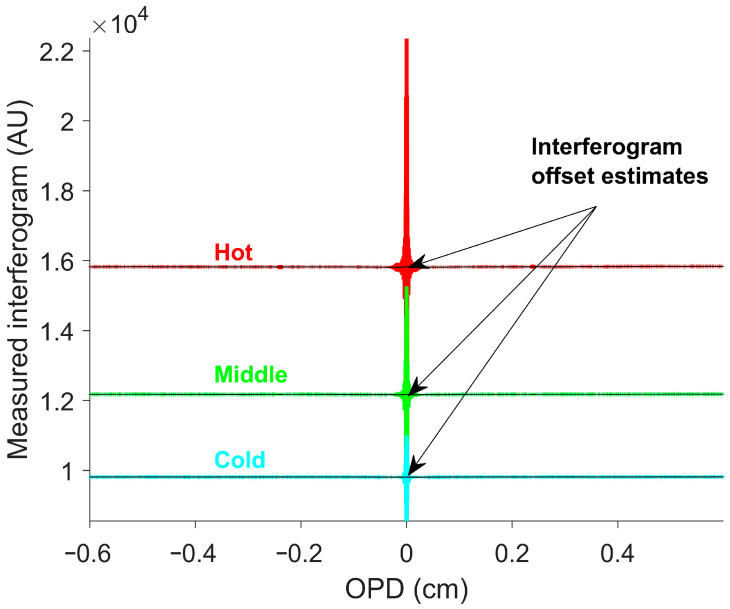
Average measured interferograms for three blackbody set temperatures. The red, green, and blue curves correspond to measured interferograms at set temperatures of 623 K, 473 K, and 373 K (350 °C, 200 °C, and 100 °C), respectively. SOC estimated the interferogram offset without significant variability near the ZPD.

For each Fluke 4181 set temperature, the measured interferograms from all 128 × 256 pixels were averaged. Measured interferograms of the reference blackbody collections were relatively flat, except for the center burst near the ZPD. Smooth offset correction (SOC) estimated the interferogram offset across its full range, including near the ZPD. Figure 11 shows the average measured interferograms for three set temperatures and the SOC-estimated interferogram offsets.

**Figure 12:**
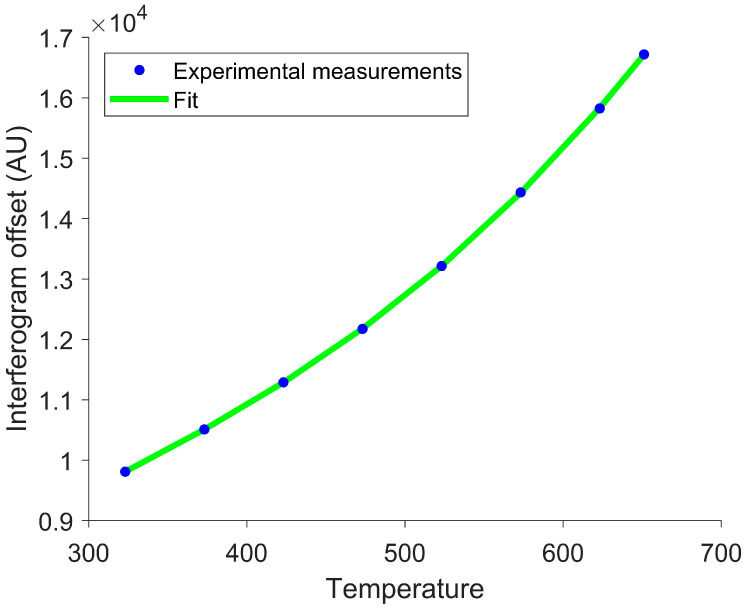
Interferogram offsets versus set temperatures. Blue circles represent the observed interferogram offset, and the green curve shows the associated fit. The polynomial fit accurately reflects the experimental measurements, suggesting that an interferogram offset can be used to determine the target’s temperature.

Interferogram offsets were subsequently averaged for each set temperature. A fourth-degree polynomial was fitted to the average interferogram offset as a function of the blackbody set temperature, as shown in Figure 12.

**Figure 13:**
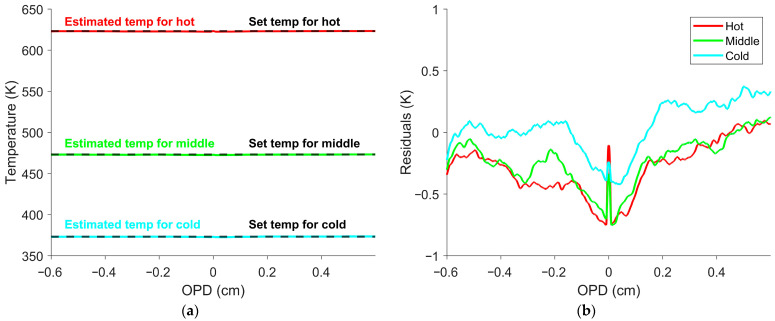
(**a**) Temperature estimates for each OPD. The red, green, and blue curves show temperature estimates at each OPD for set temperatures of 623 K, 473 K, and 373 K (350 °C, 200 °C, and 100 °C), respectively. The black curves indicate the set temperature, which were in good agreement with the estimated temperatures. (**b**) Residuals between the set temperature and its corresponding estimate. The temperature estimate was within 0.75 K of the set temperature for all OPDs.

The interferogram offset–temperature relationship was used to estimate the temperature at each OPD. Four possible temperatures resulted from solving the fourth-degree polynomial. Of the four possible solutions, three were non-physical and discarded, and the remaining solution was used as the temperature estimate. Temperatures were estimated for every OPD position and set temperature. Three temperature estimates with residuals are shown in Figure 13.

The temperature estimates in Figure 13a showed a maximum residual of 0.75 K. Although variation near the ZPD is visible in Figure 13b, SOC reduced this effect, so it is not noticeable in Figure 13a. This process was subsequently applied to each pixel. Table 3 shows the mean and standard deviation of the temperature estimates. For comparison, the mean interferograms from Figure 11 were processed to calibrated spectra and then to brightness temperatures at each wavenumber. Table 3 shows the mean and standard deviation of the brightness temperatures.

These results indicate that estimating the temperature from the measured interferogram had a comparable accuracy to that of the brightness temperature derived from the spectrum. Additionally, this temperature estimation demonstrated a higher precision, as indicated by a lower standard deviation across the OPDs compared to the standard deviation of the brightness temperature across wavenumbers. The precision reported in Table 3 was enhanced by averaging all pixels into a single interferogram prior to the temperature estimation. For individual pixels, higher variation in the temperature estimates is expected.

Drawbacks of this methodology warrant discussion. At least five calibration measurements are required, compared to the two measurements needed for two-point spectral calibration. Brightness changes in the scene will change the observed interferogram offset and then appear as changes in the target’s temperature. For example, if the blackbodies are next to the instrument during calibration and subsequently measured through 10 m of atmosphere, the additional atmosphere will reduce the brightness. For the hottest target shown Figure 13, this results in a constant temperature underestimate of 5 K. The SOC temperature estimate alone does not inform the cause of a brightness change and associates it with a temperature change. The extra atmospheric absorption is not accounted for in the calibration measurements, which increases the error of the temperature estimates. The measured spectrum may provide additional information useful for refining the SOC temperature estimate, but such refinement is not presented in this paper and has been left for future efforts. Additionally, out-of-band signals contribute to the interferogram offset, even when the calibration gain is low. Despite these limitations, this method provides a comparable accuracy, a higher precision and a significantly greater speed when estimating the target’s temperature.

### 4.2. Temperature SCA Experimental Results

**Figure 14:**
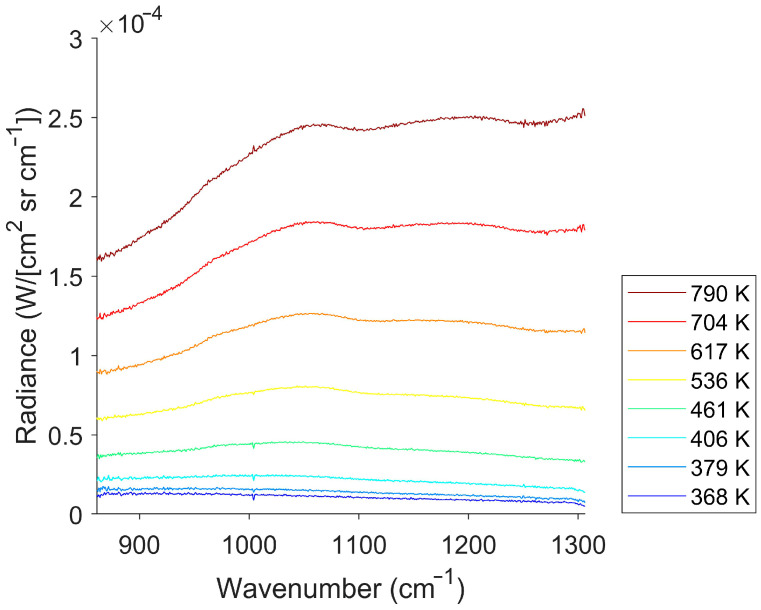
Calibrated spectra for the glow bar at steady-state temperatures. Red curves indicate higher temperatures, and blue curves indicate lower temperatures. The glow bar’s brightness temperature ranged from 368 K to 790 K (95 °C to 517 °C). While the glow bar does not mimic the performance of a blackbody, the spectrum is still relatively smooth in the detector’s response region.

In the next experiment, the glow bar acted as a target exhibiting repeatable temperature increases. At a steady state, the glow bar was measured for voltages from 1 V to 8 V, with associated currents of 0.66 A to 8.44 A. The corresponding brightness temperatures ranged from 368 K to 790 K (95 °C to 517 °C). The glow bar’s calibrated spectra are shown in Figure 14.

A “truth spectrum” is needed to evaluate the effectiveness of our correction method. The truth spectra for a given temperature will come from fitting the measured spectra in Figure 14. The spectra as a function of the input voltage were fit with a fourth-order polynomial at every wavenumber. These were then used to produce the expected spectra at any wavenumber and input voltage. A brightness temperature was determined for each expected spectrum. This then provided a truth spectrum for any brightness temperature. The average NRMSE between the estimated truth spectra and the measured spectra in Figure 14 was 0.026, indicating an imperfect fit.

**Figure 15:**
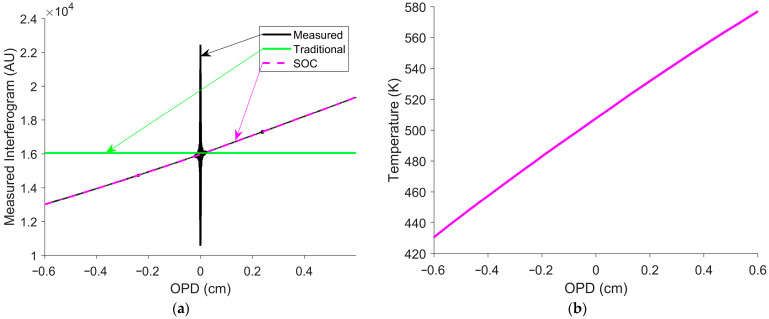
(**a**) Measured interferogram and interferogram offset estimate of the heated glow bar. The black curve indicates the measured interferogram. The green curve represents the traditional interferogram offset estimate, while the magenta curve represents the SOC interferogram offset estimate. (**b**) Temperature estimated from the interferogram offset. Estimating the interferogram offset with SOC provides a much better accuracy than the traditional constant estimate.

After cooling, 10 V and 9.5 A were applied to the glow bar. As it heated, 15 hypercubes were collected with the Hyper-Cam. The measured interferogram shown in Figure 15a, acquired during heating, represents the average of 16 pixels.

**Figure 16:**
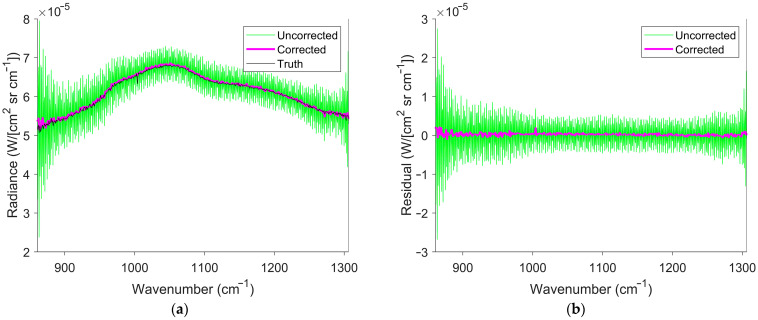
Calibrated spectra of the glow bar with and without SOC. (**a**) The green curve represents the calibrated spectrum without correction, while the magenta curve represents the calibrated spectrum with SOC. The black curve is the truth spectrum determined using the fits from Figure 14. (**b**) The green curve represents the residuals of the calibrated spectrum without correction and the truth spectrum, while the magenta curve represents the residuals of the calibrated spectrum with correction and the truth spectrum. SOC mitigates the SCAs caused by incorrect interferogram offset estimation.

The measured interferogram displayed in Figure 15a exhibited the largest change in temperature of the measured interferograms. Figure 15b shows the temperature estimated from the interferogram offset, using the techniques described in Section 2.1. The initial and final temperatures were estimated to be 429.75 K (157 °C) and 575.05 K (302 °C), respectively. The temperature at the ZPD was 506 K (233 °C). The ZPD temperature and the fits from Figure 14 were used to determine the truth spectrum. Figure 16 shows the calibrated spectra, with and without SOC, for the measured interferogram in Figure 15a.

**Figure 17:**
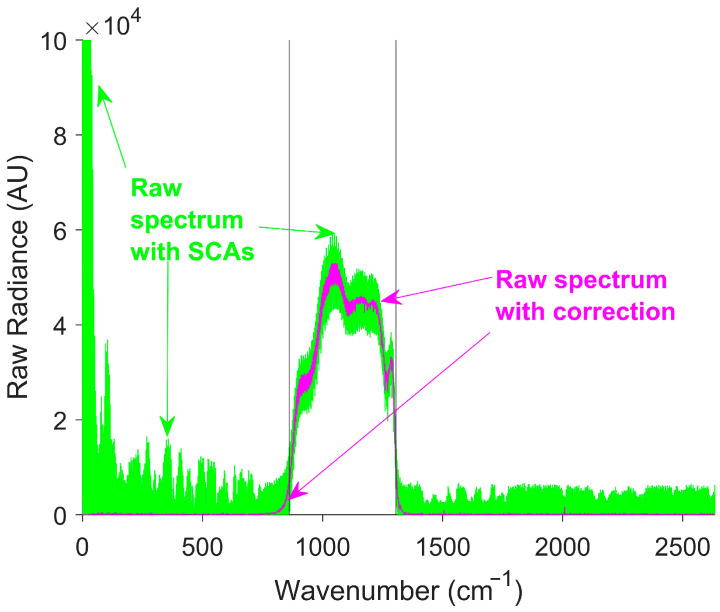
Raw spectra of the glow bar with and without SOC. The green curve represents the raw spectrum without correction, while the magenta curve represents the raw spectrum with SOC. The vertical black lines indicate the spectral response region of the LWIR Hyper-Cam. SCAs outside the detector’s response region, where the true signal is near zero, can be used to estimate the magnitude of the SCAs.

The calibrated spectrum with SOC in Figure 16 shows significant improvement compared with the spectrum without SOC. The uncorrected spectra compared to the truth spectra gave an NRMSE of 0.291. The corrected spectra compared to the truth spectra gave an NRMSE of 0.023. This is a similar NRMSE to what was seen when estimating the truth spectra using the glow bar spectra in Figure 14. The decrease in the NRMSE demonstrates significant improvement, but leaves uncertainty about the error source seen in the NRMSE of the corrected spectra. The error could originate from either an incorrect truth spectrum or from SCAs. NRMSEs could remain large even when the SCAs are eliminated. Because of this, the SSR and the raw spectrum shown in Figure 17 were used to quantify improvement in the absence of a reliable truth spectrum.

The raw spectrum without correction contained significant SCAs outside the detector’s response region. This resulted in an SSR average and standard deviation of 4±0.3 for the 16 pixels viewing the glow bar. This is due to the significant signal outside the detector response region. After SOC, the SCAs outside the response region were eliminated, improving the SSR to 89±3. Some signal still existed outside the detector response region, but this was expected, since the detector does not have a sharp cut off in its responsivity at the detector limits.

**Figure 18:**
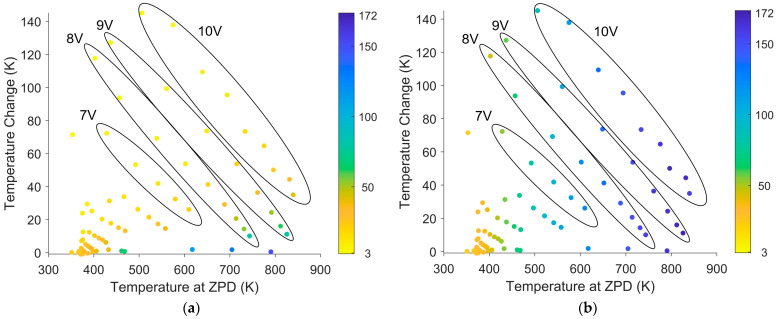
SSR versus temperature change and the ZPD temperature. (**a**) SSR without correction. (**b**) SSR with SOC. Blue circles represent a high SSR while yellow circles represent a low SSR. The large black ellipses indicate groups of measurements that were taken with the same input voltage. Without correction, the SSR decreased with a temperature change and increased with the ZPD temperature, as expected. With SOC, the SSR depended only on the ZPD temperature, since the SCAs associated with the temperature change were removed.

For each applied voltage (1–10 V), several interferograms were collected as the glow bar heated. The steady-state temperature for each applied voltage was also processed. The temperature profile was determined using the methods described in Section 2.1. The ZPD temperature and the difference between the initial and final temperatures for each interferogram were noted. Larger input voltages led to faster temperature changes and larger final temperatures. As the glow bar temperature increased, the rate of heating decreased, approaching a steady state. The measurements from different input voltages are circled in Figure 18 following this pattern. Some outliers were caused by the first interferogram of each input voltage. The FTS and glow bar were both manually activated by the experimenter. The imprecise timing meant that the glow bar began heating at different OPDs, giving rise to greater variation in the temperature change and the ZPD temperature for the first interferogram. Similarly, the SCAs and SSR saw greater variation for the first measured interferogram and associated spectrum of each run. One of these outliers is shown near 70 K of a temperature change outside the 7 V circle. Furthermore, a few high-temperature measurements were discarded due to detector saturation. The remaining interferograms were processed to raw spectra with and without correction and then to an SSR. The SSRs with and without correction are shown in Figure 18.

Larger temperatures at the ZPD generated more signal within the detector’s response region, causing the SSR to increase. When the difference between the initial and final temperatures was larger, the associated SCAs were larger, leading to a decrease in the SSR. SOC removed the SCAs caused by larger temperature differences. The result was that the SSR increased with an increasing ZPD temperature, but did not decrease with an increasing temperature change. This demonstrates that SOC was effective at mitigating temperature-variation SCAs.

## 5. Discussion

The effectiveness of SOC was demonstrated for temperature scene changes. SOC accurately estimated the interferogram offset. This included the region near the ZPD, where large signal variations challenge other methods. The interferogram offset exhibited a power-law dependence on the target temperature. This relationship enabled determination of the target temperature profile throughout the scan. A polynomial fit was used in place of the power-law relationship to account for non-ideal detector behavior. The proposed method achieved a greater temporal resolution and temperature accuracy than that achieved by using the calibrated spectrum. This improvement required only a small number of additional calibration measurements, with no other modifications to the experimental data collection.

The FTS spectral accuracy for temperature scene changes was also improved. Targets with a variable temperature exhibited corresponding variations in their measured interferograms. Measuring the temperature scene changes produced a spectrum centered on the ZPD spectrum, consistent with Kick’s prediction for FOV scene changes [15]. The interferogram variations produced SCAs superimposed on the ZPD spectrum. These multi-frequency oscillations can be difficult to distinguish from the noise in the spectrum. Approximating the variable interferogram offset as a constant introduces error, which is Fourier-transformed, producing SCAs in the spectrum. The SCA magnitude is proportional to the error between the interferogram offset and its estimate. Larger SCAs are associated with larger temperature variations, wider gain bandwidths, larger MOPDs, and a better spectral resolution. The simulations confirmed these proportionalities for both linear and exponential temperature transitions. These predictions were then validated in experiments by observing a glow bar that was heated.

SCAs arise from errors in the interferogram offset estimate and can be mitigated by improving this estimate. Applying SOC to the measured interferogram produces an accurate interferogram offset estimate. Once this step is updated, no changes are necessary to subsequent FTS processing. A new metric, the SSR, is proposed to estimate SCAs when the truth spectrum is unknown. SOC was shown to improve the NRMSE and SSR, as demonstrated through simulations and experiments when observing scene changes. Furthermore, SOC requires only a single interferogram, an improvement over other SCA correction methods, which require multiple interferograms. Previous work demonstrated that SOC effectively mitigates SCAs arising from FOV scene changes [22]. These results demonstrate that SOC mitigates SCAs from temperature scene changes as well. This is particularly important, as the similar behavior between FOV and variable-temperature SCAs can make them difficult to distinguish.

Thermal, FOV, oscillatory, and stochastic SCAs each have an associated method for mitigating SCAs. Thermal and FOV SCAs can both be mitigated by SOC. Currently, SOC has only been demonstrated for spectrally smooth blackbody targets. A future effort will investigate the technique’s effectiveness for targets with structure in the spectra. The theory presented here predicts that SOC mitigates SCAs when significant structure in the spectra is present, but this has yet to be experimentally validated. Each of the four SCA types listed above have been addressed individually. However, mitigation has not been addressed in combination. For example, a target could transition into the FOV and have a changing temperature. SOC is expected to mitigate SCAs when both artifacts occur together. If the interferogram offset is smooth, SOC should accurately estimate it, no matter the shape. For more abrupt changes, the SOC window can be reduced. For very small windows, the accuracy near the center burst is reduced. Future work will address the effectiveness of SOC on interferograms with abrupt changes to the interferogram offset.

## Figures and Tables

**Table 1:** Simulated NRMSE before and after SCA correction.

Temp Transition	NRMSE Pre-Correction	NRMSE Post-Correction
Linear	1.51	$4.34\times 10^{-3}$
Linear (LWIR)	0.12	$3.60\times 10^{-3}$
Exponential	2.09	$4.47\times 10^{-3}$
Exponential (LWIR)	0.13	$5.09\times 10^{-3}$
No transition	$1.43\times 10^{-4}$	$1.86\times 10^{-4}$

**Table 2:** SSR before and after SCA correction for simulated temperature transitions.

Temp Transition	SSR Pre-Correction	SSR Post-Correction
Linear	5.50	1160
Exponential	5.28	1120
No transition	1340	1340

**Table 3:** Temperature estimate comparison.

Set (K)	Interferogram (K)	Spectral (K)
623	622.9±0.2	623±1
473	472.9±0.2	469±1
373	373.2±0.2	370±7

## Data Availability

The datasets presented in this article are not readily available because they are not cleared for public release. Requests to access the datasets should be directed to Kody Wilson.

## Abbreviations

The following abbreviations are used in this manuscript:

FFT	Fast Fourier-transform
FOV	Field of view
FTIR	Fourier-transform infrared spectroscopy
FTS	Fourier-transform spectroscopy
IFFT	Inverse fast Fourier-transform
IR	Infrared
LOWESS	Locally weighted scatter plot smoothing
LWIR	Longwave infrared
NRMSE	Normalized root mean square error
OPD	Optical path difference
RCM	Recombobulation correction method
SCA	Scene-change artifact
SOC	Smooth offset correction
SSR	Signal-to-SCA ratio
ZPD	Zero-path difference
